# A Multiple Primary Malignancy Patient With FANCA Gene Mutation: A Case Report and Literature Review

**DOI:** 10.3389/fonc.2020.01199

**Published:** 2020-07-31

**Authors:** Qing Xia, Ling-Yi Zhao, Yi-Dan Yan, Yuan Liao, Ying-San Di, Xiu-Ying Xiao

**Affiliations:** ^1^Department of Oncology, Renji Hospital, School of Medicine, Shanghai Jiao Tong University, Shanghai, China; ^2^Department of Basic Medical Sciences, School of Medicine, Shanghai Jiao Tong University, Shanghai, China; ^3^Department of Pharmacy, Renji Hospital, School of Medicine, Shanghai Jiao Tong University, Shanghai, China; ^4^Department of Laboratory Medicine, The Third Affiliated Hospital of Sun Yat-Sen University, Guangzhou, China; ^5^Department of Oncology, Baoshan Branch Hospital, Renji Hospital, School of Medicine, Shanghai Jiao Tong University, Shanghai, China

**Keywords:** multiple primary malignancy, gastric cancer, thyroid cancer, FANCA gene, case report, literature review

## Abstract

**Background:** Multiple primary malignancies (MPMs) refer to two or more primary malignant tumors in the same individual, the prevalence of which ranges from 0. 734 to 11.7%. The risk factors for MPMs vary and include both genetic and environmental causes. FANCA gene mutation might be a predisposition to the development of a second primary cancer. Here, we report a case in which a patient with a FANCA mutation developed thyroid papillary carcinoma and gastric adenocarcinoma.

**Case Presentation:** A 48-year-old woman was diagnosed with thyroid cancer underwent resection in 2006. In 2008, the patient developed gastric adenocarcinoma and underwent radical gastrectomy. Gastric cancer was completely remitted after radiochemotherapy, but metastasis developed, and she received immunotherapy. The patient died on October 27, 2019. Peripheral blood gene detection showed germline FANCA mutation.

**Conclusions:** Gene detection is of great importance in cancer patients, especially in those with MPMs. FANCA mutation is a predisposition to tumorigenesis that can increase the risk of developing MPMs. Patients with heterozygous FANCA gene mutations have poorer outcomes.

## Introduction

Multiple primary malignancies (MPMs) refer to two or more primary malignant tumors in the same individual. The classic diagnostic criteria proposed by Warren and Gates in 1932 are as follows: (i) histologic confirmation of malignancy in both the primary and secondary tumor; (ii) the two malignancies must be anatomically separated by normal mucosa; and (iii) the possibility that the second primary malignancy is a metastasis from the first tumor must be excluded. However, there are two special conditions that should be underlined: (i) systemic cancers that could potentially involve many organs should be regarded as one, and (ii) cancers with different histology should be counted as multiple cancers, regardless of whether their sites are the same and whether the diagnoses are simultaneous (1). The prevalence of MPMs varies from 0.734 to 11.7% because of differences in geography and diagnostic approaches (2, 3). Epidemiological studies have shown that MPMs occur in the endometrium and ovary in 5% of women with endometrial cancer and in 10% of women with ovarian cancer, that the rate is 3.4% in patients with gastric cancer (GC), and that the other most common primary malignancy is colorectal cancer (20.4%) (4).

Multiple primary malignancies can be synchronous or metachronous, with diagnostic intervals of <6 months or more than 6 months, respectively. In patients with MPMs, different anticancer strategies should be applied according to the nature and stage of each tumor, making treatment challenging. In general, compared to synchronous MPM patients, metachronous MPM patients have a higher incidence and survival rate (5, 6), although curative surgery is applied more for synchronous tumors than for metachronous tumors (2).

The cooperation of both genetic and environmental factors results in cancer via the mechanisms of microsatellite instability and loss of heterozygosity. Patients with metachronous MPMs usually develop another tumor after the former one enters clinical remission, and their prognosis is poorer than that of patients with a single malignancy (7). The development of MPMs may be the result of germline mutations in cancer predisposition genes, of random chance, of radiotherapy or chemotherapy of the first tumor, or of environmental factors (8).

Herein, we report the case of a patient with a FANCA mutation who suffered from thyroid papillary carcinoma, gastric adenocarcinoma, and tumor metastasis of the ovary, posterior uterus, and gallbladder.

## Case Presentation

A 48-year-old woman was admitted for gastric malignancy on April 16, 2019, with a medical history of thyroid papillary carcinoma. In 2006, the patient underwent right thyroid cancer resection with a pathological diagnosis of thyroid papillary carcinoma, and regular follow-up visits afterward all showed complete remission. Unfortunately, she was diagnosed with gastric malignancy in 2008. The patient received radical gastrectomy in our hospital, and the pathological diagnosis was poorly differentiated adenocarcinoma of the greater curvature (diffused invasive type, 4 × 3.6 × 2 cm), and the cancer tissue had invaded into the serosa layer as well as the nerve bundle. Tumor thrombi were seen in the lymphatic vessels, whereas lymph nodes were negative. The patient was treated with radiotherapy and chemotherapy (detailed therapeutic regimen, dosage, and side effects are not available), with no obvious signs of tumor recurrence at regular follow-up.

In January 2019, she developed gradually worsening upper abdominal pain, and then nausea and vomiting occurred. However, the results of gastroscopy and colonoscopy were normal. On April 4, positron emission tomography/computed tomography indicated probable tumor metastasis by detecting a solid mass in the bilateral accessory area (58 × 55 mm on the left side, 50 × 32 mm on the right side, maximum standard uptake value was 2.7–3.1), nodules in the posterior uterus and gallbladder fossa, and increased fluorodeoxyglucose (FDG) metabolism. Abdominal pelvic, peritoneal, and pleural effusions were also detected. There were nodules at the bottom of the gallbladder with increased FDG metabolism. FDG metabolism was increased in the residual stomach and ascending and descending colon. Next-generation sequencing suggested a germline FANCA mutation, microsatellite stability, mismatch repair, and low tumor mutational burden (Table 1). The tumor cells were PD-1 [–], PD-L1 [–], PMS-2 [+], MLH-1 [+], MSH-2 [+], and MSH-6 [+].

**Table 1:** Results of peripheral blood gene detection.

**Items**	**Result**
Possible clinically significant genetic variation
FANCA	p.Q405* Exon13, 42.16%
Clinically not significant genetic variation
NOTCH2	p.T1001l, 47.91%
MAP3K1	p.W577C, 47.54%
TSC2	p.V1711M, 49.28%
Chemotherapy-related gene
UGT1A1(rs4148323)	p.G71R
UGT1A1(*6)	c.211G>A
Others
Mutation load (bTMB) (Muts count)	0
Microsatellites	MSS
HLA-1	Heterozygotic

*TMB, tumor mutational burden; MSS, microsatellite-stable; HLA, human leukocyte antigen*.

On admission, laboratory tests showed that the complete blood count, C-reactive protein level, renal function, and coagulation function were normal. However, the patient's hepatic function markedly declined, accompanied by elevated aminotransferase and bilirubin (April 17, 2019) (Table 2). Chest x-ray indicated increased lung texture with a small amount of pleural effusion on both sides. Ultrasound was used to position the ascites and showed a free anechoic zone in the abdominal cavity that was 40 mm in the deepest position and 15 mm from the body surface. The tumor marker results of ascites are listed in Table 3.

**Table 2:** Results of hepatic function.

**Items**	**April 17,**	**July 9,**	**July 31,**	**August 14,**	**Unit**	**Ref int**
	**2019**	**2019**	**2019**	**2019**		
Albumin	38.8	42.9	28	31.3	g/L	34–54
ALT	174	26	165	20	U/L	0–75
AST	261	39	174	20	U/L	10–28
TBIL	27	10	98.2	35.4	μmol/L	3.4–17.1
DBIL	17.2	4.4	82.3	28.4	μmol/L	0.1–5

*ALT, alanine aminotransferase; AST, aspartate aminotransferase; TBIL, total bilirubin; DBIL, direct bilirubin; Ref int, reference interval*.

**Table 3:** Results of ascites tumor marker detection.

**Items**	**Result**	**Unit**	**Ref int**
AFP	3.30	ng/mL	0–7
CEA	112.00	ng/mL	0–4.7
CA19-9	2,413.00	U/mL	0–27
CA125	588.00	U/mL	0–35
CA15-3	<1.50	U/mL	0–25
CA724	47.50	U/mL	0–6.9
CYFRA(21–1)	18.30	ng/mL	0–3.3
NSE	2.05	ng/mL	0–16.3
CA50	>500.00	U/mL	0–25
CA242	>200.00	U/mL	0–20

*AFP, alpha fetoprotein; CEA, carcinoembryonic antigen; CA, carbohydrate antigen; CYFRA, cytokeratins; NSE, neuron-specific enolase; Ref int: reference interval*.

Then, three cycles of chemotherapy were successfully completed from April 19, 2019, to June 14, 2019 (240 mg paclitaxel d1 + 55 mg cisplatin d1–2). On July 9, 2019, the bone scan showed multiple bone metastases. The patient received a zoledronic acid injection (4 mg) and ascites drainage (drainage of yellow skin water). On July 17, 2019, an ovarian lesion biopsy revealed pathologically that poorly differentiated adenocarcinoma infiltration or metastasis could be seen in the right ovarian tissue, indicating gastric adenocarcinoma metastasis. On July 18, 2019, the second line of chemotherapy combined with immunotherapy was administered (100 mg nivolumab d1 + 240 mg irinotecan d1 + 500 mg leucovorin d1 + 500 mg fluorouracil fast drip, 2.75 g intravenous pump maintained for 46 h). After chemotherapy, the patient experienced nausea and vomiting, followed by skin and urine yellowing and accompanied by markedly elevated aminotransferase and bilirubin, decreased albumin (Table 2), and hypokalemia (2.83 mmol/L; reference range, 3.5–5.2 mmol/L) (July 31, 2019). The abdominal magnetic resonance imaging results showed hilar occupancy with intrahepatic bile duct dilatation upstream, with a high possibility of a tumor. The patient received cholangiography and percutaneous transhepatic cholangial drainage (PTCD) on August 5. After PTCD, the patient's aminotransferase and bilirubin levels substantially improved (August 14, 2019) (Table 2). Intestinal obstruction was considered, and the condition was relieved after gastrointestinal decompression. Supportive treatments were administered during hospitalization.

On October 16, 2019, the patient was discharged with a diagnosis of stage IV gastric body carcinoma rTxNxM1 (bilateral ovaries, bone, liver, abdomen). The patient passed away on October 27, 2019. The flow chart of treatment in this patient is presented in Supplementary Figure 1.

## Discussion

Multiple primary malignancies are relatively rare in cancer patients, but the frequency of MPM occurrence has increased gradually. Many factors contribute to this increase. For example, the longer average lifetime has led to the larger elderly population, and in the general population, the development of inspection methods plays a large role (5). Additionally, the improvement of therapeutic approaches, as well as regular follow-up visits, greatly increases survival, making it possible for the development of a second primary tumor (9). Patients with metachronous MPMs usually develop another tumor after the former enters clinical remission. In general, these patients have a poorer prognosis than those with a single malignancy (7) but have a higher incidence (5) and a better prognosis (6) than synchronous MPM patients. In particular, for GC, those with MPMs have a shorter lifespan than those without MPMs (10). The treatment of MPMs is challenging because of the different nature of each primary tumor (6). One study found that, in the case of synchronous multiple primary lung cancers, surgical treatment might be a good choice (11), and most studies stress that the staging of each malignancy is the most significant factor in the decision of treatment options for MPM patients (9).

In terms of risk factors, MPMs are closely associated with male sex, old age, and the time of diagnosis of the first cancer (12). In young patients, MPMs can be attributed to genetic predisposition and the treatment of primary cancer. In adult patients, although the risk of MPMs is largely correlated with the age at diagnosis, germline mutations in cancer predisposition genes still play a significant role (8, 13). Individuals with certain germline mutations and corresponding cancer syndromes are always at high risk (14). It has been reported that most of the genes that are associated with cancer have germline mutations involved in DNA repair, and FANCA is one of these genes (15). Others are MLH1, BRCA1, BRCA2, MUTYH, ATM, PMS2, MSH6, and BAP1.

Germline FANCA mutation is the most frequent mutation in patients with Fanconi anemia (FA), an autosomal recessive inherited disease that is caused by homozygous mutation of the genes encoding Fanconi complementary group of proteins (FANCA-FANCU) (16) but is less reported in patients with solid tumors. According to The Cancer Genome Atlas database, the prevalence of somatic mutations in FANCA genes is ~3% for stomach cancer. Recently, the close relationship between alterations in FA pathways and tumorigenesis has been revealed. Hierarchical clustering analysis showed that the DNA damage repair (DDR) pathways, especially the FA pathway, were deranged across all subtypes of GC (17). The association between FANCA gene variations and breast and ovarian cancers has also been confirmed (18, 19), and the other cancers most reported to be associated with FANCA mutations are GC (17), prostate cancer (20) and colorectal cancer (21). There have been no reports on thyroid cancer yet.

Heterozygous FANCA gene mutations do not cause the FA phenotype but significantly increase cancer susceptibility in a sporadic manner (22). The major cellular function of the FA pathway is to maintain the stability of the genome during DNA replication and the damage repair process (17). Therefore, FANCA mutations will increase secondary mutations caused by DNA mismatch and the subsequent malignant transformation of normal cells, which can explain the association between FANCA mutations and the high risk of tumorigenesis, as well as, correspondingly, the association between germline mutations and an increased risk of MPM development.

The treatment of FANCA mutation carriers with malignancies should be individualized (23). A study showed that FANCA expression was associated with platinum hypersensitivity both *in vitro* and in patient-derived xenografts (24). FANCA belongs to the DDR gene, and this genetic status indicates that FA patients are more sensitive to chemotherapy, with better therapeutic efficacy but a higher rate of complications (7). On the other hand, FANCA overexpression is the major mechanism leading to cellular radioresistance, with the acceleration of DNA repair and improvement in repair fidelity, with pATM trafficking, p53/p21-axis overactivation, and so on, being the main candidate mechanism (25). Moreover, FA is associated with more than one-half of reports on adverse reactions to radiotherapy (most reported FA patients died within months of exposure) (23). Thus, cancer patients with FANCA mutations are probably hypersensitive to chemotherapy but resistant to radiotherapy. There have been no reports of treatment options for other cancers with FANCA mutations other than for breast, ovarian, and prostate cancers. Additionally, because of the rarity of studies, there are currently no Food and Drug Administration/National Medical Products Administration–approved targeting agents for FANCA gene biomarkers, and an optimum method for the prediction of radiosensitivity and the best parameter has not been found (23). For the above reasons, treatment strategies for FANCA mutation carriers depend on the specific cancer types, with consideration of predisposed MPM risk and radiotherapy-related adverse reactions.

In conclusion, in cancer patients with a diagnosis of MPM, gene detection is of great clinical significance, especially in cases of germline mutations, which increase the risk of MPM. FANCA mutation detection might be suggested in the first case of malignancy because it is a predisposition to tumorigenesis. Cancer patients with FANCA gene mutations are associated with a high risk of complications from chemoradiotherapy despite sensitivity to chemotherapy, and more clinical samples are required to further verify these findings. Therefore, supervision after therapy is extremely important in this fragile population.

## Ethics Statement

Written informed consent was obtained from the patient's next of kin for the publication of any potentially identifiable images or data included in this article.

## Author Contributions

QX, L-YZ, and Y-DY: data collection and manuscript writing. YL and Y-SD: data collection. X-YX: project development, data collection, and manuscript writing. All authors contributed to the article and approved the submitted version.

## Conflict of Interest

The authors declare that the research was conducted in the absence of any commercial or financial relationships that could be construed as a potential conflict of interest.
