# Supplementary figures and images for: A Multiple Primary Malignancy Patient With FANCA Gene Mutation: A Case Report and Literature Review
Source: Front Oncol. 2020 Jul 31;10:1199. doi: 10.3389/fonc.2020.01199 (PMC7420727; doi:10.3389/fonc.2020.01199)

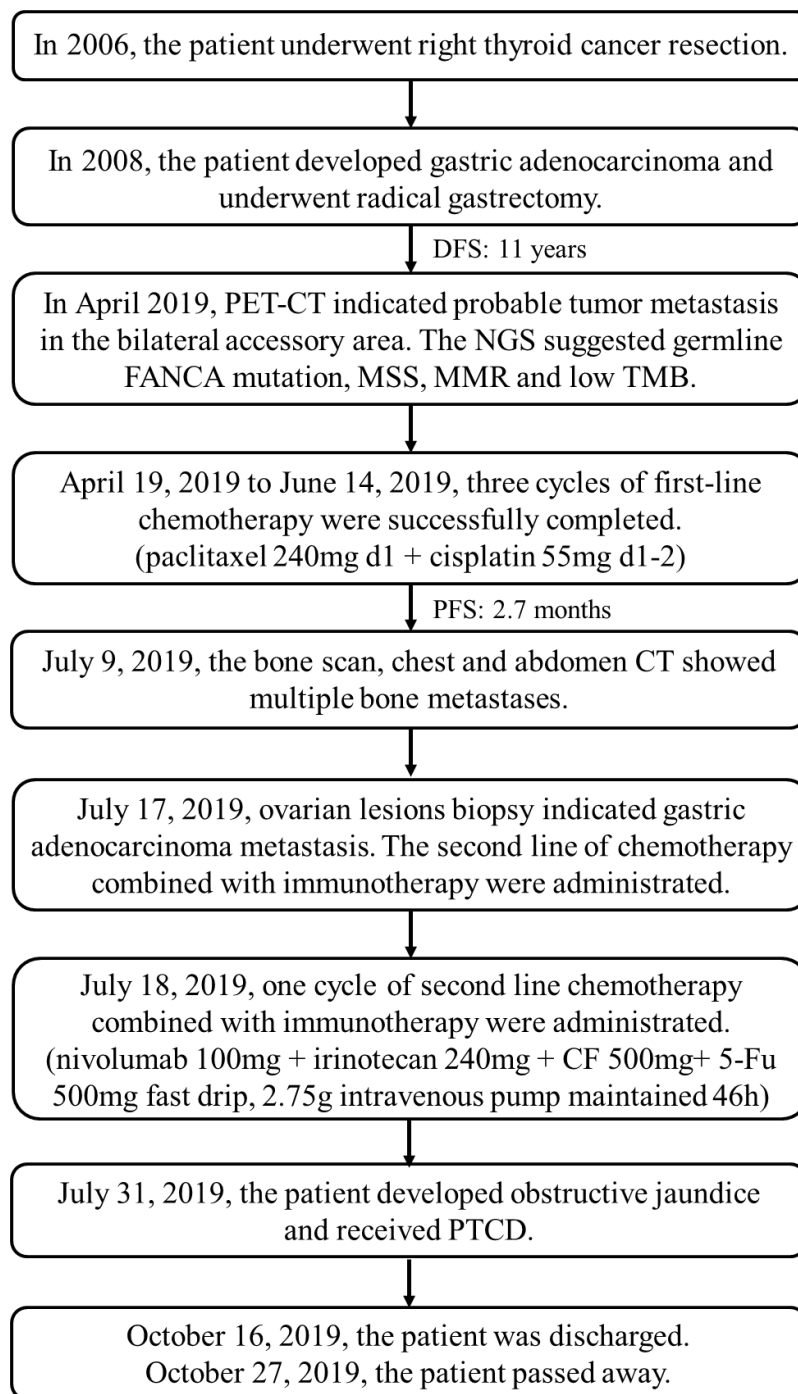

Supplementary Figure 1. Flow chart of the treatment in present patient.

Supplement: Supplementary file 1 [file Image_1.pdf]
